# Highlighting the immunohistochemical differences of malignant mesothelioma subtypes via case presentations

**DOI:** 10.1111/1759-7714.14827

**Published:** 2023-02-20

**Authors:** Anita Sejben, Tamás Pancsa, László Tiszlavicz, József Furák, Dóra Paróczai, Tamás Zombori

**Affiliations:** ^1^ Department of Pathology University of Szeged Szeged Hungary; ^2^ Department of Surgery University of Szeged Szeged Hungary; ^3^ Department of Pulmonology University of Szeged Deszk Hungary

**Keywords:** epithelioid, malignant mesothelioma, asbestos, immunohistochemistry, malignant mesothelioma, sarcomatoid malignant mesothelioma

## Abstract

Malignant mesothelioma (MM) is a rare tumor of mesothelial cells, with an increasing incidence both in developed and developing countries. MM has three major histological subtypes, in order of frequency, according to the World Health Organization (WHO) Classification of 2021: epithelioid, biphasic, and sarcomatoid MM. Distinction may be a challenging task for the pathologist, due to the unspecific morphology.

Here, we present two cases of diffuse MM subtypes to emphasize the immunohistochemical (IHC) differences, and to facilitate diagnostic difficulties. In our first case of epithelioid mesothelioma, the neoplastic cells showed cytokeratin 5/6 (CK5/6), calretinin, and Wilms‐tumor‐1 (WT1) expression, while remaining negative with thyroid transcription factor‐1 (TTF‐1). BRCA1 associated protein‐1 (BAP1) negativity was seen in the neoplastic cells' nucleus, reflecting loss of the tumor suppressor gene. In the second case of biphasic mesothelioma, expression of epithelial membrane antigen (EMA), CKAE1/AE3, and mesothelin was observed, while WT1, BerEP4, CD141, TTF1, p63, CD31, calretinin, and BAP1 expressions were not detected.

Due to the absence of specific histological features, the differentiation between MM subtypes could be a challenging task. In routine diagnostic work, IHC may be the proper method in distinction. According to our results and literature data, CK5/6, mesothelin, calretinin, and Ki‐67 should be applied in subclassification.

## Graphical abstract

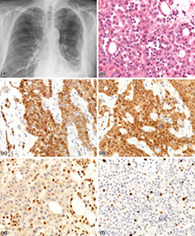

## INTRODUCTION

The first case of malignant mesothelioma (MM) was described in 1767 by Joseph Lieutand. He characterized it as “pleural tumor”, while in 1931, Rabin and Klemperer recommended the use of the term mesothelioma.^1^ MM is a rare tumor of mesothelial cells, with an increasing incidence both in developed, and developing countries. Males are 3–4 times more likely to be affected, and the average age for patients is 70 years. Few cases have been described in children, albeit in those cases, no etiological connection has been found with asbestos exposure.^2^, ^3^

This type of malignancy has high mortality due to its aggressive growth, unspecific symptoms, and difficulties in surgical removal. The pleura is by far the most commonly affected area, followed by the peritoneum, and the pericardium. Even though it is stated that MM is caused by industrial pollutants, most of all, asbestos has been defined as a causative agent, and it has also been associated with prior ionizing radiation.^4^, ^5^

Symptoms of MM are nonspecific, including dyspnea, chest pain, and general tumor manifestations, such as cachexia, fever, and fatigue. Therefore, the diagnosis is often encumbered, and delayed.^6^ Primary peritoneal mesothelioma often presents as abdominal pain, and is first misdiagnosed as cholecystitis.^7^

The first‐line diagnostic tool for MM are imaging techniques, including computed tomography (CT), magnetic resonance imaging (MRI), positron emission tomography/computed tomography (PET/CT), and ultrasonography.^8^ Despite the development of radiology, a definitive diagnosis can only be facilitated with histological evaluation.^9^ According to the World Health Organization (WHO) Classification of 2021, MM has three major histological subtypes, namely epithelioid, biphasic, and sarcomatoid.^10^, ^11^, ^12^ MM has to be distinguished from primary or secondary lung tumors, and then evidently the MM subtypes have to be differentiated from each other. Proper distinction may be a challenging task for the pathologist, due to the unspecific morphology.

The preferred treatment option for all MM subtypes is surgical resection, and a favourable outcome has been reported when combined with chemo‐ or radiotherapy, although relapse is still fairly common.^9^, ^13^, ^14^, ^15^ Regardless of the therapeutic options, the prognosis of diffuse MM remains dismal. Amin and coauthors analyzed 888 cases of pleural and peritoneal MM, and in their study, the median overall survival of these patients was 15 months, with better outcomes in patients with peritoneal involvement. Favorable prognostic factors have been identified, namely female gender, younger age (less than 45 years), epithelioid histological subtype, stage I category, peritoneal presence, and combined surgical and chemotherapeutical treatment.^16^ Sarcomatoid MM represents an even poorer prognostic group. This subtype also mainly arises from the pleura, and an association with asbestos was not found in the majority of cases examined.^17^

Two cases of MM are presented here to emphasize the clinicopathological features of this tumor focusing on immunohistochemical (IHC) characteristics, and to facilitate the establishment of correct diagnosis.

## CASE PRESENTATION 1—EPITHELIOID MM

**FIGURE 1:**
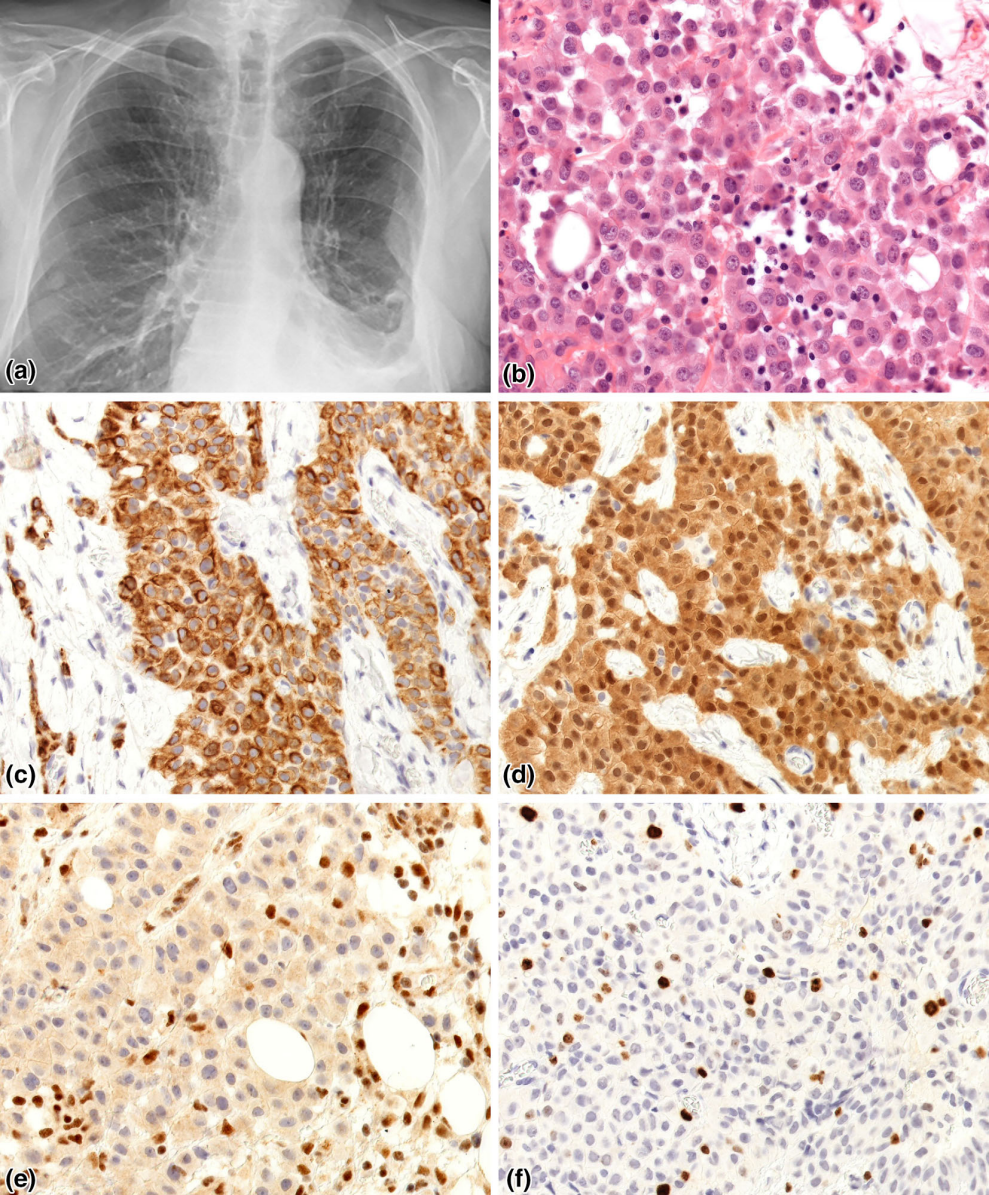
Case presentation 1—epithelioid mesothelioma. (a) Chest X‐ray examination of the patient showed signs of congestion of the pulmonary circulation, cardiomegaly and fluid accumulation in the left sinus. (b) The tumor consisted of monomorphic, epithelioid cells with severe atypia. Mild cohesion between cancer cells were visible, with small gaps among cellular interactions (HE, 40x). (c) CK5/6 IHC revealed intensive cytoplasmic positivity (CK5/6, 40x). (d) Cancer cells were block positive with calretinin staining (calretinin, 40x). (e) Alongside with positive control, the cancer cells showed expression loss of *BAP1*, proving mutation (BAP1, 40x). (f) Proliferation factor was 10% (Ki‐67, 40x)

A 78‐year‐old male patient with a history of ischemic heart disease, type 2 diabetes, and atrial fibrillation had been treated for months with therapy‐resistant hydrothorax. Even though several thoracenteses were carried out, the evaluation of the drained fluid did not confirm malignancy. In April 2021, he was admitted to the University of Szeged to surgically manage the recurring hydrothorax. During the surgery, the preliminary diagnosis was pyothorax or disseminated tumor. Chest X‐ray examination of the patient showed signs of congestion of the pulmonary circulation, cardiomegaly, and fluid accumulation in the left sinus (Figure 1a).

Macroscopic examination of the surgical specimen revealed firm, gray thickening of the pleura. Histological evaluation showed that the entire extent of the pleura had been infiltrated by relatively monomorphic, epithelioid neoplastic cells, forming solid nests or trabeculae between collagen bundles (Figure 1b). IHC was applied to identify the nature of the malignant neoplasm. The neoplastic cells showed cytokeratin 5/6 (CK5/6; Figure 1c), calretinin (Figure 1d) and Wilms‐tumor‐1 (WT1) expression, and were negative for thyroid transcription factor‐1 (TTF‐1), and BerEP4. BRCA1‐associated protein 1 (BAP1) negativity was seen in the nucleus of neoplastic cells, reflecting the loss of *BAP1* tumor suppressor genes (Figure 1e). Ki‐67 proliferation fraction was approximately 10% (Figure 1f). The case was concluded to be epithelioid MM.

Although since the surgery, the patient did not cooperate with the medical team and did not appear in the control examinations, he is still alive (overall survival: OS = 20 months).

## CASE PRESENTATION 2—BIPHASIC MM

A 69‐year‐old male patient with a history of smoking, chronic obstructive pulmonary disease, prostatic hyperplasia, and cataract was admitted to the hospital, due to an accident at home. The patient complained of severe thoracic pain, localized specifically to the ribs, and his left shoulder. During the exploration of the patient's medical history, it was discovered that he had been working as a mechanic, and although he was not known to have been exposed to asbestos, he had been heating his home with coal for a decade.

The first CT scan revealed tumorous thickening of the left sixth and seventh ribs, nearly 8 cm in largest diameter. The sixth, seventh, and eighth ribs and the intercostal muscles were surgically resected, and a GORE‐TEX patch was applied for the reconstruction of the chest wall defect.

Histological examination of the specimen showed tumorous infiltration of a biphasic neoplasm, consisting of both epithelioid, and spindle cell components. The former component formed solid nests, the latter created irregular fascicles. The atypical spindle cells demonstrated expression of epithelial membrane antigen (EMA), cytokeratin AE1/AE3 (CK AE1/AE3), and mesothelin, while WT1, epithelial cell adhesion molecule (EpCAM), thrombomodulin, TTF1, p63, CD31, and calretinin remained negative. According to the histomorphology, and immunophenotype, a rib destructing biphasic MM was diagnosed. Complete resection of the tumor could not be confirmed from the surgical specimen.

**FIGURE 2:**
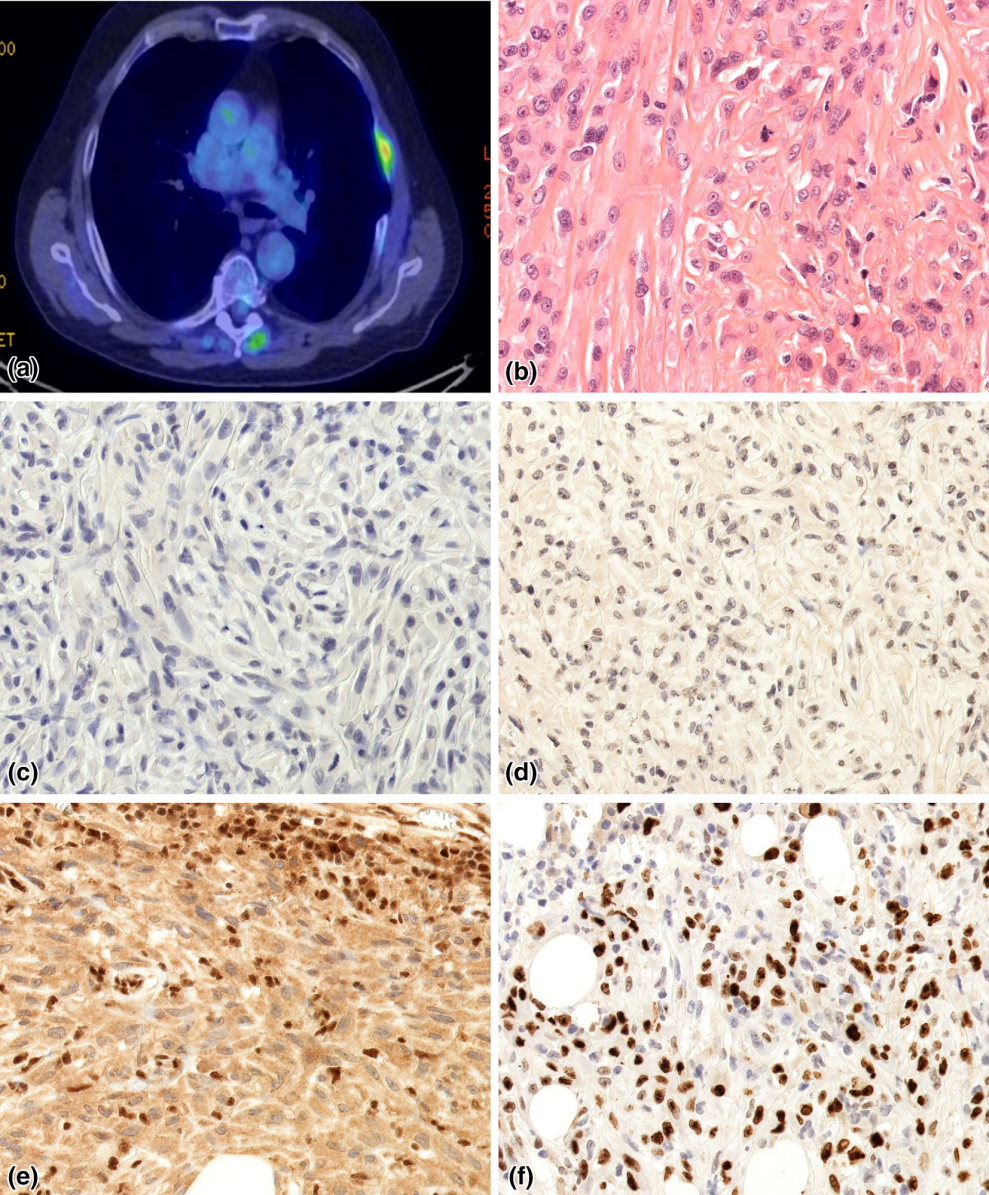
Case presentation 2—sarcomatoid mesothelioma. (a) Positron emission tomography/computed tomography (PET/CT) scan after the first surgery revealed tumorous thickening of the pleura and the ribs. (b) Spindle cell morphology cancer cells are visible, with pleomorphism. A large number of mitotic figures are also present (hematoxylin and eosin [HE], 40x). (c) CK5/6 IHC remained negative (CK5/6, 40x). (d) Cancer cells proved to be negative with calretinin staining (calretinin, 40x). (e) Loss of function mutation of BAP1 is visible (BAP1, 40x). (f) Proliferation fraction was 60% (Ki‐67, 40x)

The patient received four cycles of cisplatin, and pemetrexed combined chemotherapy. At the end of 2020, the PET/CT examination reported recurring tumorous involvement of the pleura, ribs, and also the lungs (Figure 2a). The metastatic foci located in the left upper, and lower lobes of the lung, and the infiltrated chest wall including the residual sixth rib, were removed in a second surgical procedure. The chest wall defect was covered with a GORE‐TEX patch.

Microscopic examination described tumor cells with decisively spindle cell morphology, surrounded by abundant hyalinised stroma. Focally, extreme pleomorphism, and multinucleated tumorous cells, and a large number of mitotic figures were also seen (Figure 2b). Signs of vascular, lymphovascular or perineural invasion were not present. The IHC examination revealed WT1, and mesothelin positivity in the tumor cells, while CK5/6 and calretinin remained negative (Figure 2c,d). Loss of BAP1 expression was also described, corresponding with the presence of mutant *BAP* gene (Figure 2e). Mitotic rate was high (21 mitoses/10 high power fields). Ki‐67 proliferation marker was expressed in 60% of tumor cells (Figure 2f). The results of IHC examination, and the microscopic morphology ratified the diagnosis of sarcomatoid MM. Alongside the GORE‐TEX patch, severe foreign body reaction developed, with chronic inflammation, and numerous giant cells.

Novel tumorous infiltration of the basis of the left lung has been reported in the most recent control PET/CT examination. The third surgical procedure will be performed with video‐assisted thoracic surgery, and is due in the near future. The OS of the patient is currently 24 months.

Because complete resection of the tumor from the first surgery was not proven, it can be stated that the tumor developed as biphasic MM from the beginning, and later the more aggressive part with sarcomatoid morphology recidivated. The OS also supports this hypothesis. Even though the patient was not known to have been exposed to asbestos, the literature already describes the association of MM with coal.^18^

## DISCUSSION

Albeit histologically MM can show diverse morphology, prognosis correlates well with the epithelioid, biphasic, and sarcomatoid classification.^12^ According to the study by Amin et al., the median survival of the epithelioid subtype was 18 months, while it proved to be 10 months for the biphasic subtype, and remained only 7 months for the sarcomatoid subtype.^16^ In a large scale series by Brustugun and coworkers that examined 1509 MM cases in a 20‐year‐long period, an even worse prognosis was observed, with median survival of 5.1 months for nonepithelioid subtypes.^19^

The chemotherapeutical response of MM subtypes has been investigated in some studies. In the meta‐analysis by Mansfield et al., the results of 41 trials were analyzed and revealed that the rate of response to chemotherapy was only 21.9%, and 13.9% of patients with epithelioid, and sarcomatoid MM, respectively.^11^

A primary diagnosis of MM is still demanding. Differential diagnosis includes primary lung adenocarcinoma, squamous cell carcinoma, sarcomatoid carcinoma, vascular tumors, melanoma, and metastatic origin (breast, gastrointestinal, prostate, kidney, ovary, thyroid cancer etc) also has to be excluded. Less frequent, but possible challenging diagnosis constitute lymphomas, *SMARCA4‐*deficient thoracic tumors, desmoplastic small round cell tumor, monophasic synovial sarcoma, and *CIC*‐translocated sarcomas. Regarding nontumorous conditions, inflammation, chronic pyothorax, reactive mesothelial hyperplasia, pleuritis, and callus must be considered.^20^

Regarding differential diagnosis of diffuse MM, Ali and coauthors introduced a pattern‐based approach in 2018. Regarding reactive pleural changes versus diffuse MM, the application of the following IHC markers are favored.^21^ Desmin and glucose transporter 1 (GLUT1) remains generally positive in mesothelial hyperplasia.^22^ In cases of p53, aberrant or nonwild‐type expression could serve as a clue in comprehending malignant versus benign lesions.^23^ EMA positivity has been linked both to reactive and neoplastic lesions, although its combined use with desmin could serve as a solution, while EMA positivity alongside with desmin negativity favors diffuse MM. Its opposite, EMA negativity and desmin positivity facilitates reactive processes.^24^, ^25^ Positivity of insulin‐like growth factor II messenger ribonucleic acid binding protein‐3 (IMP‐3), and thrombomodulin IHC markers tend to be observed more in diffuse MM cases, rather than those which are reactive.^26^, ^27^

Differentiation of chronic, active, fibrosing pleuritis may be difficult, as a result of the misinterpretation of fatlike spaces being present in organizing pneumonia, and pleuritis cases to real fat tissue infiltration (stromal infiltration) of desmoplastic MM. In such scenarios, S100 can help in discerning actual fat tissue, and fatlike structures. In most cases, the discrimination is mainly based on examination of the hematoxylin and eosin (HE) staining, because a laminar appearance has to be present in fibrosing pleuritis. From inside to outside, several layers have to be defined, including fibrin, neutrophil granulocytes, mononuclear inflammatory cells, granulation tissue, and connective tissue composed of hyalinised collagen bundles.^21^

Somatic mutation of tumor suppressor gene, *BAP1* has been described as fairly common in diffuse MM. The loss of *BAP1* can be observed in the majority of epithelioid, and mixed (60–70%), while it is present in 15% of sarcomatoid MM cases. Since the mutation results in protein loss, during IHC examination, BAP1 negativity could be seen. The lack of BAP1 expression has a low sensitivity (20%–53%), but approximately a 100% specificity as a marker of diffuse MM, therefore BAP1 can serve as a useful tool for distinguishing MM from reactive lesions.^21^, ^28^

According to the results of Ali et al., fluorescent in situ hybridization (FISH) could be useful in selected cases in order to differentiate benign, and malignant lesions. Since *CDKN2A* gene codes two proteins via alternative splicing (p16/INK4A and p14/ARF), its loss is detectable. Although this examination has 100% specificity for the diagnosis of MM, it is not sufficient for differentiating epithelioid, and sarcomatoid subtypes.^21^ Further molecular diagnostic procedures have not yet been described. Methylthioadenosine phosphorylase (MTAP) is a newly described IHC surrogate of FISH.^29^

According to the recommendations of the current WHO, in cases of distinction of carcinoma versus epithelioid, and mixed MM subtypes, at least two carcinoma and two mesothelial IHC markers are required, due to their low sensitivity.^12^, ^30^, ^31^ Spindle cell malignancies can be differentiated from sarcomatoid mesothelioma, with calretinin and D2‐40.^21^

Even after finally agreeing upon a diagnosis of MM, the histological evaluation of MM subtypes could also be a challenging task for pathologists because of their nonspecific morphology; therefore, conducting IHC can help in confirming the final diagnosis.^32^ In compliance with the above mentioned sections, the following diagnostic algorithm can be applied in cases of epithelioid MM. After exclusion of reactive processes, carcinomas, and mesenchymal neoplasms, additional EMA, desmin, IMP‐3, and thrombomodulin positivity can be observed in the majority of cases, alongside with BAP1 loss.

On the other hand, sarcomatoid MM tends to be negative with WT1, B cell lymphoma‐2 (Bcl‐2), CD34, and desmin. In light of the results of Chirieac et al., the majority of sarcomatoid MM cases showed either negativity, or focal positivity of keratin markers, including CKAE1/AE3, CAM 5.2, and MNF 116. Solely, one fourth of cases were positive with calretinin.^33^

The review by Rossi et al. highlights the possible aberrant expression of several markers including p40 (5,5%) and p63 positivity in epithelioid MMs, as well as the positivity of the TTF1 SP141clone in 42% of sarcomatoid MM cases.^34^ Husain and coauthors emphasize that there is currently no useful IHC recommendation on this matter, furthermore, in some cases, no positivity could be observed, due to the overfixation of the surgical specimen.^35^

We would like to further illustrate the diagnostic challenges of MM by mentioning the reproducibility examinations previously reported.

The first dates back to 1997 when five pathologists evaluated 77 cases of HE staining, and later evaluated the cases with IHC markers, including cytokeratins, vimentin, HMFG‐2, CD15, BerEP4, B72.3, and carcinoembyonic antigen (CEA). The results reflect that IHC did not change the diagnosis of MM in most cases.^36^

Brčić et al. focused on the differentiation of MM subtypes. Three pathologists assessed 200 MM cases, one representative HE slide from each, and moderate agreement (κ = 0.36) was achieved at the first round, while substantial agreement (κ = 0.63) was observed in the second round, after a consensus meeting. The authors emphasize the use of a strict, consensus based diagnosis.^37^

A diagnosis of biphasic mesothelioma possibly remains the hardest task after all. Based on the reproducibility examination of by the International Mesothelioma Panel from the MESOPATH Reference Center, moderate interobserver correlation was achieved (weighted **κ** = 0.45), with 14 examiners evaluating 544 cases by using only BAP1 and p16 IHC stainings.^38^

Our two cases and Table 1 summarize the most commonly used and worldworld widely available IHC markers for the differentiation of MM subtypes. Mutual positivity was observed with WT1, and mutual negativity was seen with TTF‐1 or napsin‐A, excluding the possibility of primary lung cancer. In both subtypes, BAP1 was negative, reflecting the loss of gene expression. The most helpful markers in our cases proved to be CK5/6, mesothelin, calretinin, and Ki‐67. The epithelioid subtype showed positivity with all of them, and Ki‐67 proliferation marker was 10%. On the other hand, the sarcomatoid subtype remained negative with CK5/6 and calretinin, had focal cytoplasmic positivity with mesothelin, and Ki‐67 proliferation marker was 50%–60%. We recommend the use of these widely available markers.

**TABLE 1:** Synthesis of the immunohistochemical (IHC) differences of epithelioid and sarcomatoid mesothelioma

IHC marker	Epithelioid mesothelioma	Sarcomatoid mesothelioma
CK5/6	+ (cytoplasmic)	‐
WT1	+ (nuclear)	+ (nuclear)
Mesothelin	+ (diffuse) membranous and cytoplasmic	+ (focal) cytoplasmic
Calretinin	+ (block)	‐
BAP1	‐/loss of expression	−/loss of expression
Ki‐67	10%	50%–60%

## CONCLUSIONS

The differentiation between MM subtypes could be a challenging task, due to the lack of specific histological features. IHC may be the optimal method in distinction. WT1, TTF‐1, BAP1 markers help setting the diagnosis of MM, while CK5/6, mesothelin, calretinin, and Ki‐67 are helpful in the establishment of subclassification.

## AUTHOR CONTRIBUTIONS

Concept and design – Anita Sejben, Tamás Zombori, Tamás Pancsa. Search and evaluation of references – all authors. Drafting the manuscript – Anita Sejben, Tamás Zombori. Approval of final manuscript – all authors.

## CONFLICT OF INTEREST

The authors declare no conflict of interest.

## ETHICS APPROVAL STATEMENT

Ethics approval statement was provided from the Institutional Committee of the Albert Szent‐Györgyi Medical University.

## Data Availability

The authors confirm that the data supporting the findings of this study are available within the article.
